# Residual Risks of Thrombotic Complications in Anticoagulated Patients with Atrial Fibrillation: A Cluster Analysis Approach from the GLORIA-AF Registry

**DOI:** 10.1007/s11606-024-09045-6

**Published:** 2024-09-25

**Authors:** Hironori Ishiguchi, Azmil H. Abdul-Rahim, Bi Huang, Steven Ho Man Lam, Yang Liu, Brian Olshansky, Tze-Fan Chao, Menno V. Huisman, Gregory Y. H. Lip, on behalf of the GLORIA-AF Investigators

**Affiliations:** 1 Liverpool Centre for Cardiovascular Science at University of Liverpool, Liverpool John Moores University and Liverpool Heart & Chest Hospital, Liverpool, UK; 2 Division of Cardiology, Department of Medicine and Clinical Science, Yamaguchi University Graduate School of Medicine, Ube, Japan; 3 Department of Cardiovascular and Metabolic Medicine, Institute of Life Course and Medical Sciences, University of Liverpool, Liverpool, UK; 4 Stroke Division, Department Medicine for Older People, Whiston Hospital, Mersey and West Lancashire Teaching Hospitals NHS Trust, Prescot, UK; 5 Division of Cardiology, Department of Internal Medicine, University of Iowa Hospitals and Clinics, Iowa City, USA; 6 Division of Cardiology, Department of Medicine, Taipei Veterans General Hospital and National Yang Ming Chiao Tung University, Taipei, Taiwan; 7 Department of Thrombosis and Hemostasis, Leiden University Medical Center, Leiden, The Netherlands; 8 Danish Centre for Health Services Research, Department of Clinical Medicine, Aalborg University, Aalborg, Denmark

**Keywords:** ischaemic stroke, cluster analysis, anticoagulation therapy

## Abstract

**Background:**

Assessment of residual thromboembolic risk in patients with atrial fibrillation (AF) prescribed oral anticoagulants (OACs) remains unexplored. We performed hierarchical cluster analysis to identify phenotypic profiles of these patients and their risks of residual thromboembolic events.

**Methods:**

We utilised data from non-valvular AF patients on OACs, as documented in phases II and III of the GLORIA-AF (Global Registry on Long-Term Oral Anti-thrombotic Treatment in Patients With Atrial Fibrillation) registry. We performed a hierarchical cluster analysis to identify distinct phenotypic profiles. We compared the incidence and risks of thromboembolic events (composite of ischaemic stroke, transient ischaemic attack, or systemic embolism) and related outcomes (major bleeding and all-cause death) across the profiles. We determined the optimal number of profiles through visual inspection of the generated dendrograms.

**Results:**

We included 22,410 patients (mean age 70 ± 8 years; 56% male), from which five phenotypes were identified: profile 1 (“uncontrolled hypertension”), profile 2 (“young with a history of coronary artery disease”), profile 3 (“young and obese”), profile 4 (“frailty”), and profile 5 (“non-paroxysmal AF with tachycardia”). Profile 4 was associated with the highest rates of thromboembolic events (1.66/100 person-years [95% confidence interval, 1.46–1.89]), major bleeding (1.92/100 person-years [1.70–2.16]), and death (6.02/100 person-years [5.62–6.43]). Profile 3 was associated with the lowest risk across all measured outcomes (thromboembolic events, 0.64 events/100 person-years [0.48–0.82]; major bleeding, 0.83 events/100 person-years [0.65–1.04]; and death, 1.44 events/100 person-years [1.21–1.71]). Profile 1 had a moderate thromboembolic event rate (1.04/100 person-years [0.91–1.08]), while profiles 2 and 5 showed lower rates.

**Conclusions:**

The phenotypic profiles of patients with AF prescribed OACs identified using hierarchical cluster analysis are associated with distinct residual thromboembolic risks and related outcomes. This approach has the potential to enhance patient risk-stratification and holistic approaches to management.

**Graphical Abstract:**

AF, atrial fibrillation; CAD, coronary artery disease; F/U, follow-up; HTN, hypertension; IS, ischaemic stroke; NVAF, nonvalvular atrial fibrillation; OACs, oral anticoagulants; PAF, paroxysmal atrial fibrillation; SE, systemic embolism; TE, thromboembolism; TIA, transient ischaemic attack

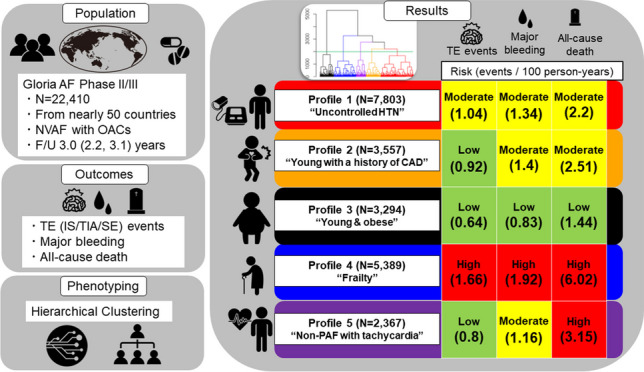

**Supplementary Information:**

The online version contains supplementary material available at 10.1007/s11606-024-09045-6.

## Introduction

Oral anticoagulant (OAC) therapy is the cornerstone in the management of patients with atrial fibrillation (AF), reducing the risk of ischaemic stroke by two-thirds.^1^ Increased adherence to clinical guidelines has enhanced the prescription rates of OACs among at-risk patients, reflecting a positive trend in clinical practice.^2,3^

Despite judicious use of OACs, a residual risk of ischaemic stroke remains evident.^4^ Real world data from a globally diverse cohort of patients with AF showed an annual incidence of stroke of 0.6%.^5^ Thromboembolic risk stratification in this population is complex; risk factors include, but are not limited to, advanced age, diabetes mellitus and chronicity, history of thromboembolic events, and lack of antiarrhythmic drug usage.^4,5^ Indeed, many risk factors do not occur in isolation, and tend to cluster, leading to clinically complex patient scenarios with implications for treatments and prognosis.^6–8^ Such clinical complexity reflects the heterogeneity inherent in the AF development and maintenance.^9^

Cluster analysis represents a relatively new unsupervised machine learning/statistical technique that segregates populations into distinct clusters based on common phenotypes.^10^ Previous studies have identified several phenotypes within patients with AF, associated with unique risk profiles for clinical events.^11–13^ However, the generalisability of these findings across a global, ethnically diverse population remains uncertain due to the geographically limited scope of most studies.

To address this issue, we assessed the residual risk of thromboembolic events among patients with AF on OACs within the GLORIA-AF (Global Registry on Long-Term Oral Antithrombotic Treatment in Patients With Atrial Fibrillation) registry, which spans a diverse multi-ethnic cohort of AF patient population. By employing hierarchical cluster analysis, we aimed to identify phenotypic profiles and their risks of residual thromboembolic events, in order to improve future risk stratification.

## Methods

We utilised data from phase II and III of the GLORIA-AF registry, a prospective, international, multicentre registry programme designed to gather the demographic data and outcomes on recently diagnosed non-valvular AF patients at risk of stroke across approximately 50 countries.^14^ Briefly, the inclusion criteria were as follows: (1) age ≥ 18, (2) non-valvular AF diagnosed within 3 months (extended to 4.5 months for Latin America populations) from the baseline visit, and (3) CHA_2_DS_2_-VASc score ≥ 1. Exclusion criteria included AF attributed to a reversible cause, anticipated or history of mechanical heart valve surgery, extensive prior use of vitamin K antagonists (VKA), clinical indications for OACs other than AF, and a prognosis of limited life expectancy (< 1 year). The enrolment period was 2011–2014 for phase II and 2014–2016 for phase III. Follow-up was typically 2 years for participants who were prescribed dabigatran in phase II and 3 years for those prescribed any OACs in phase III. Ethical approval was obtained from the institutional review boards of all participating centres, with all participants providing written informed consent. The study adhered to the principles of the Declaration of Helsinki and was conducted in accordance with Good Clinical Practice guidelines.

### Study Outcomes

The primary outcome of this study was the incidence of thromboembolic events among patients receiving OACs. These composite events included ischaemic stroke, transient ischaemic attack (TIA), or systemic embolism. The clinical diagnosis of ischaemic stroke and TIA was confirmed through computed tomography or magnetic resonance imaging.^15,16^ Systemic embolism was defined as an acute vascular occlusion of the extremities or any organ, typically diagnosed by angiography, surgery, scintigraphy, or autopsy.^15^

The secondary outcomes for this study were incidence of major bleeding events and all-cause mortality. Major bleeding was defined according to the International Society of Thrombosis and Haemostasis criteria, which included significant haemoglobin reduction (> 20 g/L), the requirement for transfusion (> 2 units of blood or packed cells), symptomatic bleeding in a critical organ, or bleeding that is life-threatening or results in death.^6,17^

### Hierarchical Cluster Analysis

We constructed a hierarchical cluster using the “dendextend” package in R, focusing on patient demography, which included 43 variables. Initially, we calculated the Euclidean distances between patient cases. This method measures the straight-line distance between points in a multidimensional space, providing a clear and intuitive understanding of the similarities or differences between cases.^13^ This step allows us to create a distance matrix that serves as the foundation for hierarchical clustering.

Subsequently, we applied Ward’s minimum variance method to evaluate and minimise within-cluster variance. This approach resulted in a dendrogram that visually represents the clustering of patients based on their demographic data.^11^

These variables included age, sex, body mass index (BMI), systolic/diastolic blood pressure (BP), pulse rate, European Heart Rhythm Association class, AF type (paroxysmal AF [PAF], persistent/permanent AF), duration of AF, creatinine clearance, type of OACs, region, ethnicity, smoking status, and alcohol consumption status, along with a comprehensive list of comorbid conditions. These comorbidities included hypertension, hyperlipidaemia, diabetes, coronary artery disease (CAD), congestive heart failure (CHF), previous stroke/TIA/systemic embolism, peripheral artery disease, chronic obstructive pulmonary disease, dementia, hyperthyroidism, cancer, and chronic gastrointestinal disease. We also included AF-specific therapies such as ablation, cardioversion, and left atrial appendage occlusion, along with medications like angiotensin-converting enzyme inhibitors (ACEI), angiotensin II receptor blockers, beta blockers, verapamil, diltiazem, digoxin, statins, diuretics, oral hypoglycaemic agents, insulin, and antiarrhythmic drugs.

For handling missing data and outlier values, we employed the Multiple Imputation by Chained Equations technique. We determined the optimal number of phenotypic profiles through visual assessment of dendrograms, balancing the average silhouette width with the size of each cluster.

### Statistical Analysis

Variables with normal distributions were represented as mean ± standard deviation, whereas those with non-normal distributions were presented as medians along with the first and the third interquartile. The analysis of variance was utilised for comparing these variables. Categorical variables were expressed as numbers and percentages and compared using the chi-square test. Kaplan–Meier curves delineated event-free survival for each outcome. Differences among clusters were compared using the log-rank test. The incidence rate of each outcome was expressed as the number of events per 100 person-years, accompanied by a 95% confidence interval (CI). The hazard ratio (HR) for each outcome, along with its 95% CI, was calculated using a univariate Cox proportional hazards model. The cluster (i.e. phenotypic profile) with the lowest incidence was designated as the reference. Results were considered statistically significant at a *p*-value of less than 0.05. All statistical analyses were conducted using R version 4.0.4.

## Results

### Patient Demographics of Each Profile

**Figure 1:**
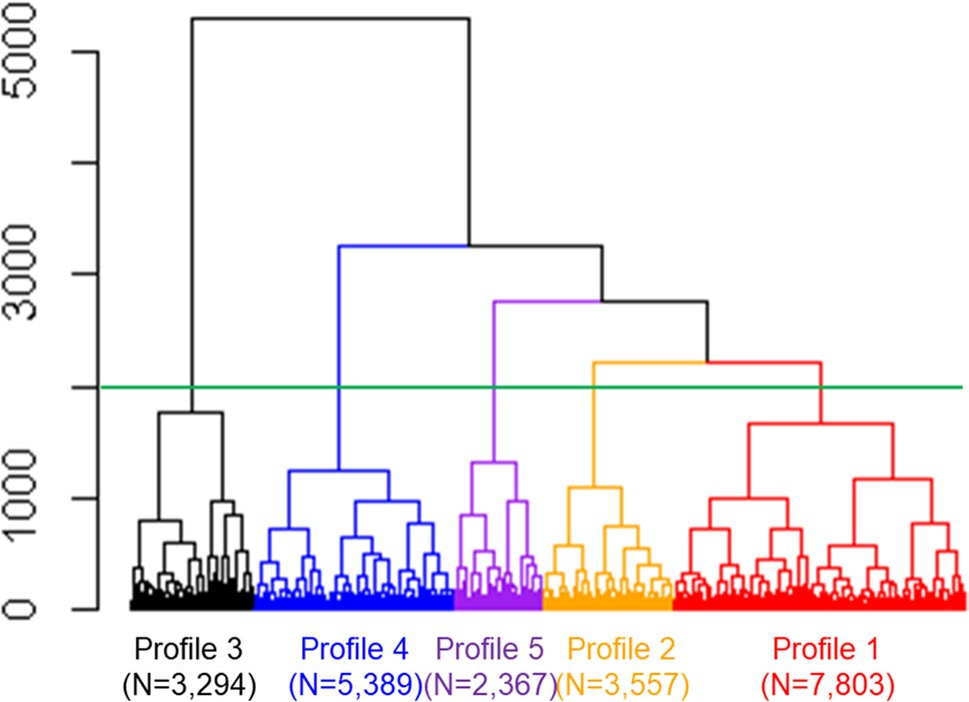
Dendrogram obtained from hierarchical clustering. The horizonal line indicates the cut-off line the whole cohort dividing 5 profiles.

Among 36,617 patients eligible for the GLORIA-AF Phase II/III cohort, 22,410 patients who took OACs and for whom information on thromboembolic events was available were included (Supplementary Fig. 1). We constructed a dendrogram for this population, as illustrated in Fig. 1. Based on the visual clarity from the dendrogram and the numerical evaluation (Supplementary Table 1), we selected the configuration with five profiles, as it offered a fair balance of average silhouette width and cluster distribution. The demographics characteristics of each profile are summarised in Table 1.

**Table 1:** Patient Demographics

	Profile 1 (*N* = 7803)	Profile 2 (*N* = 3557)	Profile 3 (*N* = 3294)	Profile 4 (*N* = 5389)	Profile 5 (*N* = 2367)	*P*-value
Age, years, mean ± SD [1.5]	71 ± 8	69 ± 8	60 ± 8	78 ± 7	71 ± 9	< 0.0001
Sex, male, *n* (%) [0]	4386 (56)	2115 (59)	2290 (70)	2354 (44)	1221 (52)	< 0.0001
BMI (kg/m^2^), mean ± SD [2.4]	29 ± 5	28 ± 5	34 ± 6	26 ± 4	29 ± 5	< 0.0001
Systolic BP (mmHg), mean ± SD [1.5]	143 ± 14	114 ± 9	132 ± 17	127 ± 16	135 ± 19	< 0.0001
Diastolic BP (mmHg), mean ± SD [1.5]	82 ± 10	70 ± 9	81 ± 12	73 ± 10	83 ± 11	< 0.0001
Pulse rate (/min), mean ± SD [2.2]	72 ± 13	78 ± 15	79 ± 16	75 ± 14	117 ± 13	< 0.0001
EHRA Class, mean ± SD [6.3]	1.9 ± 0.9	2.0 ± 0.9	1.9 ± 0.9	1.9 ± 0.9	2.1 ± 0.9	< 0.0001
AF duration (months), median (IQR) [0]	2.3 (0.4, 5.7)	2.4 (0.6, 6)	2.7 (0.6, 6.1)	2.1 (0.6, 5.6)	0.7 (0.1, 3.3)	< 0.0001
Creatinine clearance (ml/min), mean ± SD [20]	79 ± 17	81 ± 14	139 ± 28	46 ± 13	80 ± 25	< 0.0001
CHADS_2_, mean ± SD [0.01]	1.9 ± 1.1	1.6 ± 1.1	1.6 ± 0.9	2.3 ± 1.2	1.8 ± 1.1	< 0.0001
CHA_2_DS_2_-VASc, mean ± SD [0]	3.2 ± 1.4	2.9 ± 1.4	2.3 ± 1.2	4.0 ± 1.4	3.2 ± 1.4	< 0.0001
HAS-BLED, median (IQR) [10]	1 (1, 2)	1 (1, 2)	1 (0, 1)	1 (1, 2)	1 (1, 2)	0.002
Antithrombotic agents, *n* (%) [0]						< 0.0001
VKA	1609 (21)	787 (22)	623 (19)	1321 (25)	496 (21)	
Dabigatran	3219 (41)	1324 (37)	1284 (39)	1905 (35)	990 (42)	
Apixaban	1469 (19)	728 (20)	641 (19)	1193 (22)	474 (20)	
Rivaroxaban	1363 (17)	673 (19)	724 (22)	888 (16)	367 (16)	
Edoxaban	143 (2)	45 (1)	22 (1)	82 (2)	40 (2)	
Region, *n* (%) [0]						< 0.0001
North America	1520 (19)	975 (27)	1153 (35)	1110 (21)	373 (16)	
Europe	4446 (57)	1590 (45)	1577 (48)	2713 (50)	1572 (66)	
Latin America	593 (8)	290 (8)	159 (5)	646 (12)	107 (5)	
Africa/Middle East	104 (1)	47 (1)	65 (2)	50 (1)	48 (2)	
Asia	1140 (15)	655 (18)	340 (10)	870 (16)	267 (11)	
Ethnicity, *n* (%) [7.3]						< 0.0001
Black/Afro-Caribbean	131 (2)	60 (2)	85 (3)	90 (2)	41 (2)	
Asian	978 (13)	630 (18)	260 (8)	848 (16)	263 (11)	
White	6318 (81)	2659 (75)	2787 (85)	4113 (76)	1946 (82)	
Arab/Middle East	112 (1)	51 (1)	73 (2)	55 (1)	53 (2)	
Others	264 (3)	157 (4)	89 (3)	283 (5)	63 (3)	
Smoking status, *n* (%) [3.4]						< 0.0001
Never smoked	4739 (61)	1974 (55)	1650 (50)	3503 (65)	1490 (63)	
Current smoker	643 (8)	378 (11)	490 (15)	283 (5)	237 (10)	
Ex-smoker	2421 (31)	1205 (34)	1154 (35)	1603 (30)	640 (27)	
Alcohol drinking status, *n* (%) [6.3]						< 0.0001
No alcohol	3328 (43)	1557 (44)	1126 (34)	2887 (54)	1107 (47)	
< 1 drink/week	2165 (28)	870 (24)	933 (28)	1362 (25)	615 (26)	
1–7 drinks/week	1681 (22)	831 (23)	871 (26)	918 (17)	452 (19)	
≥ 8 drinks/ week	629 (8)	299 (8)	364 (11)	222 (4)	193 (8)	
AF type, *n* (%) [0]						< 0.0001
Paroxysmal AF	4441 (57)	1886 (53)	1762 (53)	2869 (53)	928 (39)	
Persistent AF	2527 (32)	1316 (37)	1279 (39)	1755 (33)	1170 (49)	
Permanent AF	835 (11)	355 (10)	253 (8)	765 (14)	269 (11)	
Medical history, *n* (%)						
Hypertension [0.2]	6270 (80)	2302 (65)	2678 (81)	4102 (76)	1785 (75)	
Hyperlipidaemia [2.7]	3330 (43)	1472 (41)	1445 (44)	2269 (42)	844 (36)	< 0.0001
Diabetes [0]	1778 (23)	757 (21)	1039 (32)	1183 (22)	528 (22)	< 0.0001
CAD [2.6]	1349 (17)	705 (20)	470 (14)	1204 (22)	339 (14)	< 0.0001
CHF [0.8]	1331 (17)	921 (26)	740 (23)	1468 (27)	607 (26)	< 0.0001
History of stroke/TIA/systemic embolism [0]	1259 (16)	458 (13)	365 (11)	1016 (19)	248 (11)	< 0.0001
Previous bleeding [0.7]	408 (5)	179 (5)	129 (4)	341 (6)	88 (4)	< 0.0001
PAD [0.8]	235 (3)	90 (3)	65 (2)	232 (4)	60 (3)	< 0.0001
COPD [1.1]	430 (6)	253 (7)	189 (6)	383 (7)	145 (6)	0.0004
Dementia [1.2]	25 (0.3)	16 (0.4)	3 (0.1)	78 (1.4)	10 (0.4)	< 0.0001
Hyperthyroidism [1.5]	207 (3)	98 (3)	73 (2)	170 (3)	61 (3)	0.12
Cancer [1.4]	758 (10)	351 (10)	219 (7)	671 (13)	246 (10)	< 0.0001
Chronic gastrointestinal disease [1.4]	993 (13)	503 (14)	429 (13)	799 (15)	240 (10)	< 0.0001
Previous AF therapy, *n* (%)						
Previous AF ablation [0]	136 (2)	75 (2)	91 (3)	69 (1)	37 (2)	0.0001
Previous cardioversion [0]	1406 (28)	710 (20)	813 (25)	777 (14)	400 (17)	< 0.0001
Previous LAAO [0]	8 (0.1)	5 (0.1)	2 (0.1)	5 (0.1)	0	0.45
Other agents, *n* (%)						
Antiplatelet agents [0]	1294 (17)	659 (19)	562 (17)	1059 (20)	350 (15)	< 0.0001
ACEI [0]	2538 (33)	1103 (31)	1281 (39)	1532 (28)	712 (30)	< 0.0001
ARB [0]	2290 (29)	730 (21)	800 (24)	1491 (28)	609 (26)	< 0.0001
Beta-blockers [0]	4813 (62)	2324 (65)	2270 (69)	3344 (62)	1683 (71)	< 0.0001
Verapamil [0]	89 (1)	42 (1)	57 (2)	67 (1)	42 (2)	0.03
Diltiazem [0]	377 (5)	237 (7)	285 (9)	296 (6)	143 (6)	< 0.0001
Digoxin [0]	475 (6)	352 (10)	285 (9)	473 (9)	351 (15)	< 0.0001
Statins [0]	3579 (46)	1603 (45)	1445 (44)	2509 (47)	914 (39)	< 0.0001
Diuretics [0]	2902 (37)	1312 (37)	1310 (40)	2441 (45)	960 (41)	< 0.0001
Oral hypoglycaemic [0]	1182 (15)	489 (14)	769 (23)	692 (13)	347 (15)	< 0.0001
Insulin [0]	299 (4)	131 (4)	192 (6)	261 (5)	98 (4)	< 0.0001
AADs, antiarrhythmic drugs [0]	2039 (26)	966 (27)	913 (28)	1337 (25)	656 (28)	< 0.0001

Numerical data are expressed as mean ± SD or median (interquartile range; first quartile, third quartile). Categorical data are expressed as percentages and numbers. [] indicate missing rate (%)

*ACEI* angiotensin-converting enzyme inhibitor, *AF* atrial fibrillation, *ARB* angiotensin II receptor blocker, *BMI* body mass index, *BP* blood pressure, *CAD* coronary artery disease, *CHF* congestive heart failure, *COPD* chronic obstructive pulmonary disease, *EHRA* European Heart Rhythm Association, *IQR* interquartile range, *LAAO* left atrial appendage occlusion, *PAD* peripheral artery disease, *SD* standard deviation, *TIA* transient ischaemic attack, *VKA* vitamin K antagonist

Profile 1, characterised by “uncontrolled hypertension”, showed the highest mean systolic BP at 143 ± 14 mmHg and a prevalent hypertension rate of 80%. Notably, this profile also had the highest incidence of cardioversion history at 28%. The profile predominantly composed of individuals of white ethnicity (81%).

Profile 2, identified as “young with a history of CAD”, has a relatively youthful average age of 69 ± 8 years. This group has the highest history of CAD at 20% and the most extensive use of antiplatelet agents at 19%, with the exception of the “frailty” profile. It also has the lowest recorded systolic and diastolic BP (114 ± 9 mmHg and 70 ± 9 mmHg, respectively), and had the largest representation of Asian ethnicity (18%).

Profile 3, the “young and obese” group, was the youngest cohort with an average age of 60 ± 8 years and predominantly male (70%). This profile was noted for its high BMI of 34 ± 6 kg/m^2^, the highest creatinine clearance at 139 ± 28 ml/min, and the lowest CHADS_2_ and CHA_2_DS_2_-VASc scores. Predominantly of White ethnicity (85%) with significant proportion from North America (35%), this profile also had the highest percentages of current smokers and alcohol consumers, alongside with the highest prevalence of atherosclerotic risk factors including hypertension, hyperlipidaemia, and diabetes.

Profile 4, or the “frailty” cluster, was characterised by an advanced average age of 78 ± 7 years and majority female (56%). It had the lowest BMI and creatinine clearance values, alongside the highest CHADS_2_ and CHA_2_DS_2_-VASc scores, reflecting a high burden of comorbidities such as CAD, CHF, and previous stroke/TIA/systemic embolism. This profile also showed the highest intake of VKA at 25% and considerable representation from Latin America (12%) and Asia (16%).

Profile 5, labelled as “non-PAF with tachycardia”, was noted for the highest pulse rate at 117 ± 13/min and a 61% prevalence of persistent/permanent AF. This group had high rates of CHF, second only to the profile 4 (“frailty”), and the highest usage of beta blockers, digoxin, and antiarrhythmic drugs. Majority of its participants were from the European region (66%).

### The Incidence for Thromboembolic Events

**Figure 2:**
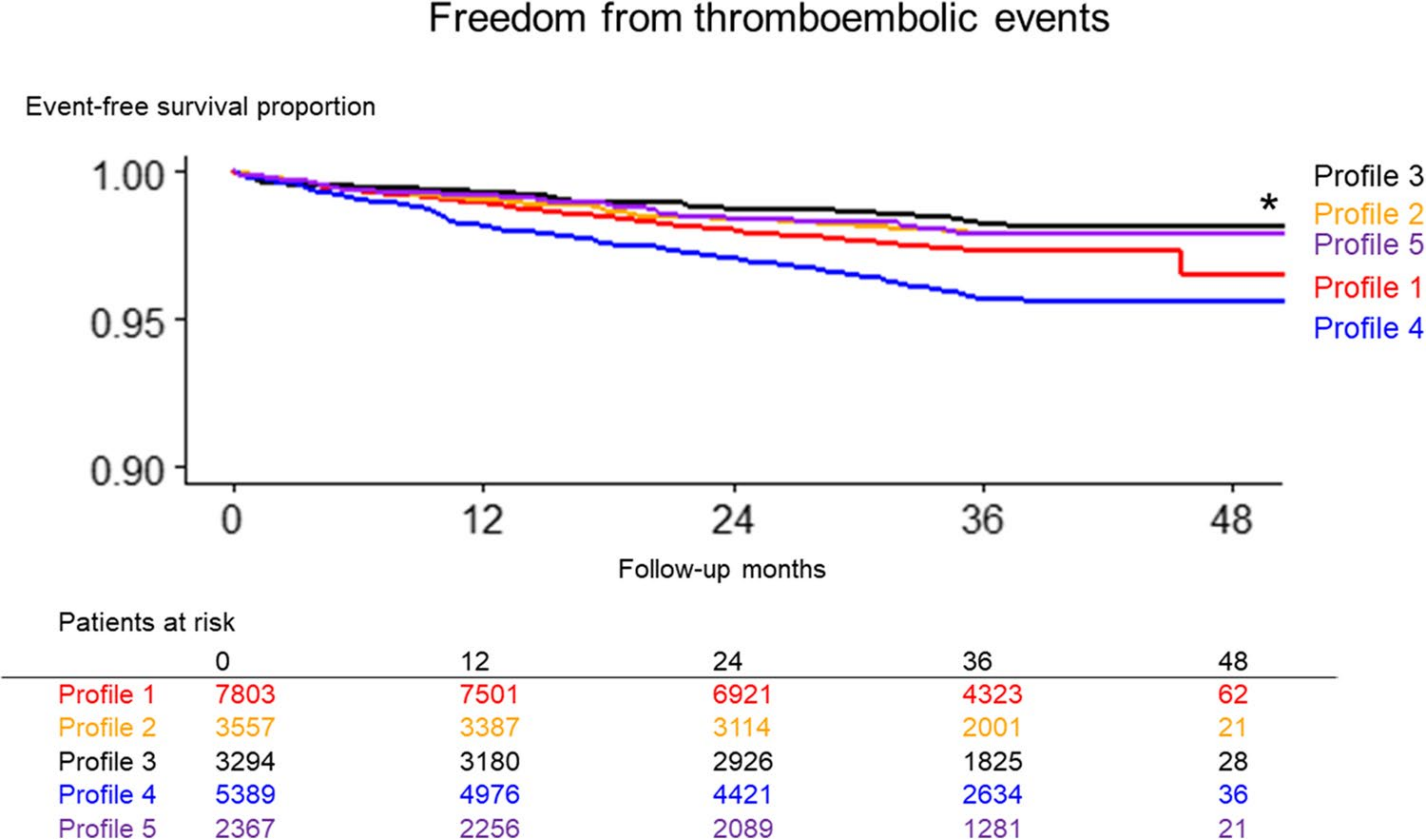
The Kaplan–Meier curve comparing the incidence of thromboembolic events across 5 profiles. Asterisk indicates statistical significance (*P* < 0.001).

During the study period, with a median follow-up of 3.0 (IQR, 2.2–3.1) years, 598 patients had thromboembolic events: ischaemic stroke at 60%, TIA at 32%, and systemic embolism at 8%. The differential risk across the profiles was depicted through a Kaplan–Meier curve analysis (Fig. 2), which highlighted the highest cumulative incidence within profile 4 (“frailty”) and the lowest in profile 3 (“young and obese”).

Profiles 2 (“young with a history of CAD”), 3 (“young and obese”), and 5 (“non-PAF with tachycardia”) showed relatively low thromboembolic incidence rates (less than 1 event per 100 person-years). Conversely, profiles 1 (“uncontrolled hypertension”) and 4 (“frailty”) showed rates ranging from moderate to high (Fig. 5 [left panel] and Table 2). The risk of thromboembolic events in profiles 1 (“uncontrolled hypertension”) and 4 (“frailty”) was significantly higher, compared to the profile 3 (“young and obese”), with HRs 1.54 (95% CI, 1.13–2.09) and 2.46 (95% CI, 1.81–3.34), respectively (Fig. 6 [left panel] and Table 3).

**Table 2:** Incidence Rates for each Clinical Event Across Profiles

	Profile 1	Profile 2	Profile 3	Profile 4	Profile 5
TE, /100 person-years (95% CI)	1.04 (0.91–1.18)	0.92 (0.74–1.13)	0.64 (0.48–0.82)	1.66 (1.46–1.89)	0.80 (0.60–1.05)
Major bleeding, /100 person-years (95% CI)	1.34 (1.19–1.5)	1.40 (1.17–1.65)	0.83 (0.65–1.04)	1.92 (1.70–2.16)	1.16 (0.91–1.45)
All-cause death, /100 person-years (95% CI)	2.20 (2.01–2.41)	2.51 (2.20–2.84)	1.44 (1.21–1.71)	6.02 (5.62–6.43)	3.15 (2.73–3.61)

*TE* thromboembolism

**Table 3:** Hazard Ratios for each Clinical Event Across Clusters

	Profile 1	Profile 2	Profile 3	Profile 4	Profile 5	*P*-value
TE, HR (95% CI)	1.54 (1.13–2.09)	1.22 (0.85–1.75)	Reference	2.46 (1.81–3.34)	1.19 (0.79–1.78)	< 0.0001
Major bleeding, HR (95% CI)	1.69 (1.28–2.22)	1.70 (1.25–2.30)	Reference	2.42 (1.84–3.19)	1.48 (1.05–2.08)	< 0.0001
All-cause death, HR (95% CI)	1.54 (1.27–1.88)	1.76 (1.42–2.18)	Reference	4.36 (3.61–5.26)	2.22 (1.78–2.78)	< 0.0001

*HR* hazard ratio, *TE* thromboembolism

### The Incidence for Major Bleeding

**Figure 3:**
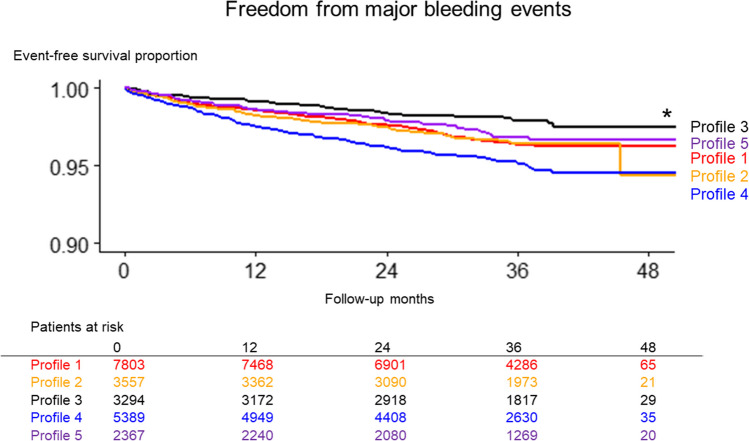
The Kaplan–Meier curve comparing the incidence of major bleeding across 5 profiles. Asterisk indicates statistical significance (*P* < 0.001).

During the study period, 775 patients had major bleeding events. Kaplan–Meier curve analysis, depicted in Fig. 3, highlighted the disparate risk levels across the profiles, with profile 4 (“frailty”) having the highest cumulative incidence of major bleeding, and profile 3 (“young and obese”) having the lowest. This distribution of risk for major bleeding mirrored the trends observed for thromboembolic events.

The incidence rates for major bleeding among profiles 1 (“uncontrolled hypertension”), 2 (“young with a history of CAD”), 3 (“young and obese”), and 5 (“non-PAF with tachycardia”) were comparably steady, with values ranging from 1.2 to 1.4 events per 100 person-years (Fig. 5 [middle panel] and Table 2). In contrast to profile 3 (“young and obese”), the other profiles showed significantly higher risk of major bleeding, HRs 1.69 (95% CI, 1.28–2.22) for profile 1, 1.70 (95% CI, 1.25–2.3) for profile 2, 2.42 (95% CI, 1.84–3.19) for profile 4, and 1.48 (95% CI, 1.05–2.08) for profile 5 (Fig. 6 [middle panel], Table 3).

### The Incidence for All-Cause Death

**Figure 4:**
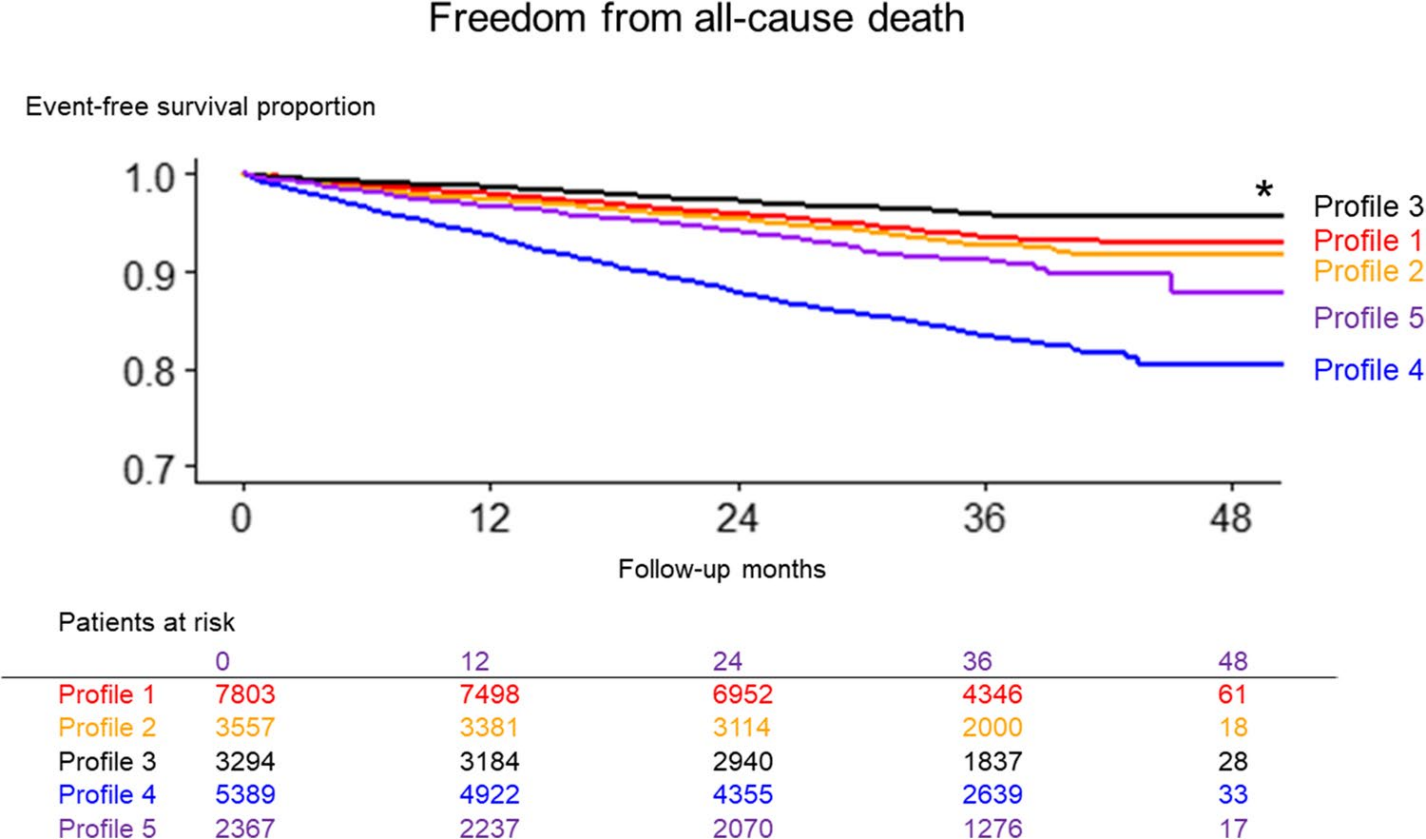
The Kaplan–Meier curve comparing the incidence of all-cause death across 5 profiles. Asterisk indicates statistical significance (*P* < 0.001).

During the study period, 1918 all-cause deaths were documented. Kaplan–Meier curve analysis (Fig. 4) highlights the variation in mortality risk across different profiles. Profile 4 (“frailty”) had the highest incidence of mortality, while profile 3 (“young and obese”) had the lowest, a trend consistent with the patterns observed for other outcomes.

**Figure 5:**
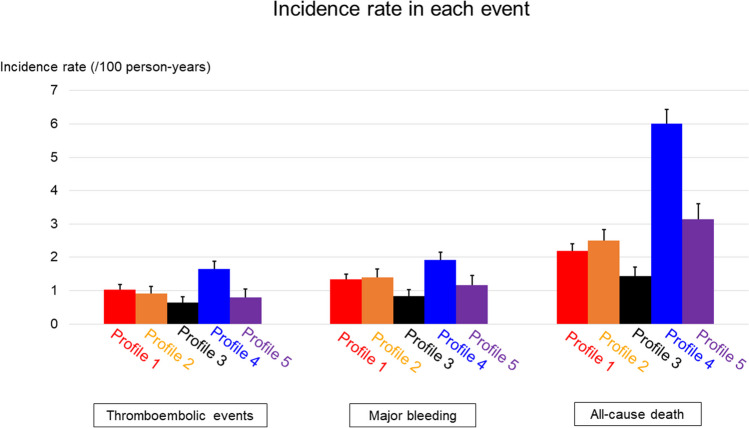
The comparison of incidence rate of each event across 5 profiles. Each bar indicates the incidence rate (/100 persons-year) with 95% confident interval.

**Figure 6:**
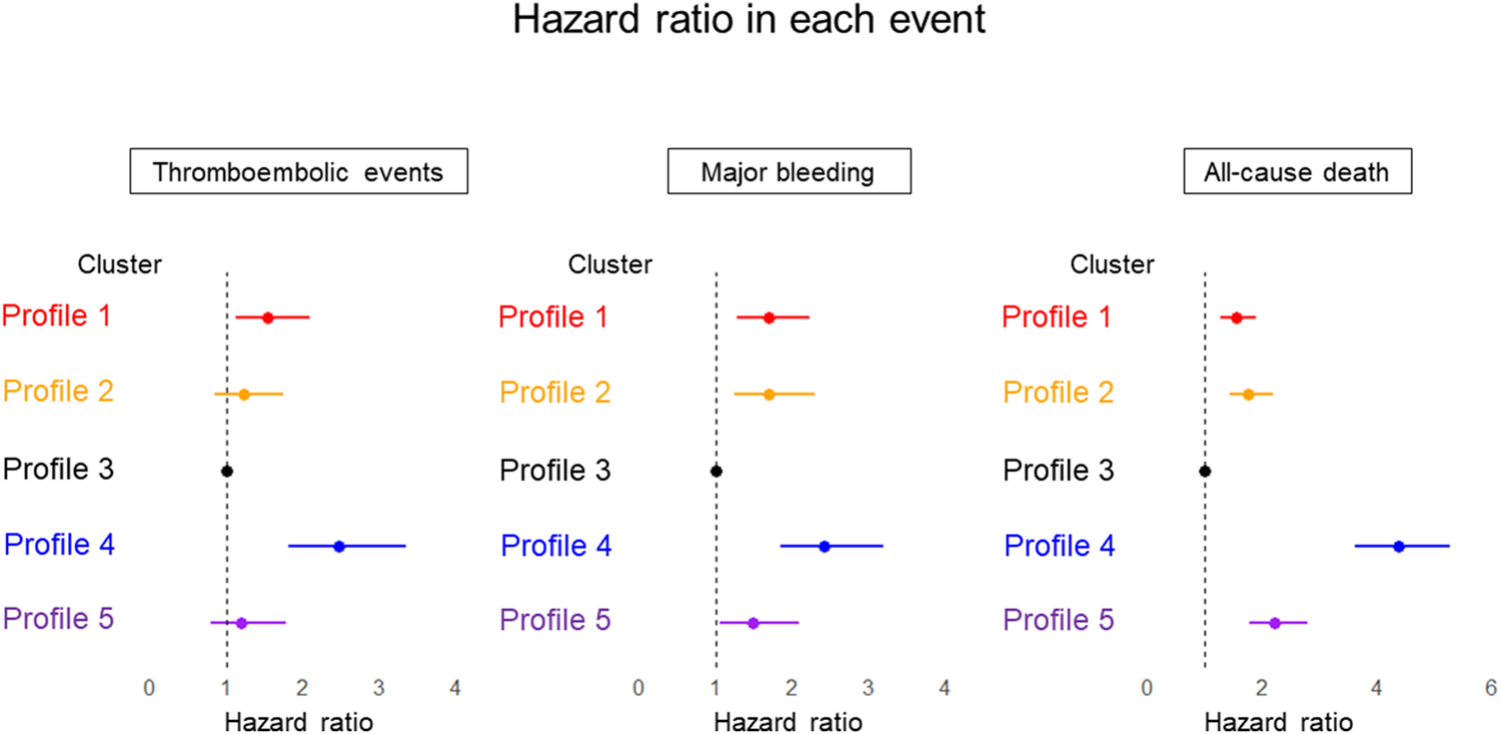
The comparison of hazard ratio of each event across 5 profiles. Each plot indicates the hazard ratio with 95% confident interval. Profile 3 was defined as reference.

The annual incidence rates for mortality in for profiles 1 (“uncontrolled hypertension”) and 2 (“young with a history of CAD”) were comparable, at 2.2 and 2.5 events per 100 person-years, respectively. In contrast, profile 5 (“non-PAF with tachycardia”) demonstrated a higher rate of 3.2 events per 100 person-years (Fig. 5 [right panel] and Table 2). Compared with profile 3 (“young and obese”), a significantly increased mortality risk was observed in the other profiles: HRs 1.54 (95% CI, 1.27–1.88) for profile 1, 1.76 (95% CI, 1.42–2.18) for profile 2, 4.36 (95% CI, 3.61–5.26) for profile 4, 2.22 (95% CI, 1.78–2.78) for profile 5 (Fig. 6 [right panel], Table 3).

## Discussion

The key findings of our study can be summarised as follows. Through hierarchical cluster analysis, we were able to stratify the residual thromboembolic risks in patients with AF who already prescribed OACs. Within the delineated clusters, profile 4 (“frailty”) was associated with the highest risk across all outcomes, including thromboembolic events, major bleeding, and all-cause death. In contrast, profile 3 (“young and obese”) showed the lowest risk for these outcomes. Profile 1 (“uncontrolled hypertension”) showed a moderate risk for residual thromboembolic events. Meanwhile, profiles 2 (“young with a history of CAD”) and 5 (“non-PAF with tachycardia”) were associated with low risk levels. Finally, despite the low to moderate risk for other outcomes, profile 5 (“non-PAF with tachycardia”) was significantly associated with an increased risk of all-cause death (Graphical Abstract).

### The Characteristics in High-Risk and Low-Risk Phenotypes

Previous research has tried to stratify patients with AF according to distinct risk profiles.

These classifications differ based on selected variables, criteria for inclusion or exclusion, and the demographic characteristics of the study populations. Nonetheless, a recurring differentiation emerges: a “high-risk phenotype” characterised by clinically-important comorbidity burden and an older age demographic, and a “low-risk phenotype”, marked by fewer comorbidities and a younger patient demographic.^11–13,18^

Building on these insights, our analysis identifies profile 4 (“frailty”) and profile 3 (“young and obese”) that are similar to the high-risk and low-risk phenotypes within the spectrum. Previous work with the GLORIA-AF cohort identified a “clinical complexity” subgroup. This group, defined by older age, low BMI, chronic kidney disease, and/or a history of bleeding, was associated with an increased risk of clinical events and a lower rate of OACs prescriptions.^6^ While our current study is limited to patients receiving OACs, the risk profile of the “clinical complexity” subgroup closely parallels that of profile 4 in our results. Our findings introduce the novel insight that this phenotype continues to present an increased risk for adverse events, even when managed with anticoagulation therapy.

Our findings highlights the association between the obese phenotype and relatively favourable outcomes, adding depth to the discourse on the “obesity paradox”.^19^ The epigenetic factors that mediate the crosstalk between environmental conditions and genetic predisposition can contribute to this phenomenon by expressing specific genes that have a protective effect in response to obesity.^20,21^

The pattern of phenotypes with lower event risk correlating with higher BMI was observed across other cohorts.^11,13^ Our study provides novel insights by highlighting the predominance of this phenotype particularly in the North American among individuals of White ethnicity, as opposed to other regions and ethnic backgrounds. Despite the “young and obese” profile (profile 3) had a higher prevalence of atherosclerotic risk factors compared to others, it nonetheless exhibits a lower risk of adverse events. This is consistent with previous study which showed while these risk factors increase the rate of adverse events in normal-weight patients, the same correlation does not hold for overweight individuals.^22^ This discrepancy might be attributed to overweight patients receiving early and more intensive treatment interventions, as evidenced by higher administration of therapeutic agents such as ACEIs and oral hypoglycaemic agents.^22^ It is also plausible that profile 3 in the present study also reflects this pattern as well. In addition, the epigenetic factors which mediate the crosstalk between environmental condition and genetic predisposition can contribute “obesity paradox” by expressing specific genes which function protective effect under the obese condition.

### The Importance of Uncontrolled Hypertension for Residual Thromboembolic Risk

Our analysis identified a moderate residual risk of thromboembolic events in patients with uncontrolled hypertension. Hypertension is well recognised as a risk factor for stroke.^23^ However, recent studies suggested that the risk associated with hypertension may be more intricately linked to the effectiveness of BP management than merely to hypertension’s status.^24–26^ Even among AF patients on anticoagulation therapy, a longitudinal cross-sectional study suggested that a systolic BP level exceeding 140 mmHg is associated with an increased residual risk of thromboembolic events compared to those with well-controlled hypertension.^27^ Our results are aligned with this body of evidence.

### Clinical Implications

Our study highlights the phenotypic differences among patients with AF who are on OACs from a global, diverse population, including nearly 50 countries worldwide with more than 20,000 patients. Compared to previous studies addressing phenotypic differences using hierarchical cluster analysis, our population allows for the evaluation of risk stratification and regional/ethnic differences among the profiles obtained. We highlight the clinical implications of our study as follows.

Our findings highlighted management considerations certain phenotypes, emphasising uncontrolled hypertension as a crucial marker of increased risk for residual thromboembolic events. Moreover, our data support the critical role of good BP management for the phenotype, as studies have demonstrated that a proactive approach in reducing BP can markedly reduce the incidence of major vascular events, including stroke, in patients with AF.^28^

Furthermore, our data emphasised the need for individualised therapeutic strategies that extend beyond simple anticoagulation for profiles 4 (“frailty”) and 5 (“non-PAF with tachycardia”). An integrated and holistic approach, known as the Atrial fibrillation Better Care (ABC) pathway^29^—that is, Avoidance of stroke, Better symptom management, and Cardiovascular risk and comorbidity optimisation—is essential for a more holistic or integrated care management of these complex patient profiles. Adherence to the ABC pathway is associated with a reduction in adverse outcomes, including all-cause mortality,^30,31^ leading to its recommendation in guidelines.^32^ A recent study have shown that patients with high-risk profiles stand to gain greater benefits from the integrated therapeutic approach compared to those with low-risk profiles.^13^ The phenotypic profiles we presented could enhance risk-stratification and aid in identifying potential good candidates for this holistic therapeutic approach.

The impact of regional and ethnic differences within each phenotype also merits consideration. For example, profile 3 (“young and obese”) predominantly observed in North American, while profiles 1 (“uncontrolled hypertension”) and 5 (“non-PAF with tachycardia”) were more common in Europe, mostly among individuals of White ethnicity. In contrast, individuals with Asian ethnicity are more frequently associated with profiles 2 (“young with a history of CAD”) and 4 (“frailty”). These data would facilitate better understanding of the influence of ethnicity and regional variations in relation to patient’s risk profiles and outcomes.

### Limitations

Our study has limitations. First, our hierarchical cluster analysis primarily utilised basic demographics data and did not include anatomical or functional parameters, such as left atrial volume and left ventricular ejection fraction. However, the phenotyping approach, based on readily accessible data, offers simplicity for clinical application. Second, our analysis was restricted to patients prescribed OACs. This may limit the generalisability of our findings to the wider AF population. For example, the representation of Asian ethnicity within profile 4 (“frailty”) may be underrepresented due to a predisposition towards lower OAC use among frail patients in this group.^33^ Third, our study involved a diverse population and 43 variables, highlighting the uncertainty surrounding the optimal sample size for clustering methods, as power calculations for these methods are not well-established in the literature. Despite this, we believe our sample size of 22,410 patients is appropriate, allowing for fair stratification.

Fourth, the study cohort’s ethnic diversity was limited, notably lacking individuals with Black or Arab/Middle Eastern ethnicity. Fifth, the methods of hierarchical clustering are susceptible to errors such as overfitting and sensitivity to noise, which are inherent to unsupervised machine learning. The results can be influenced by the choice of distance metrics and linkage criteria, potentially leading to different interpretations. Moreover, the concept of the “black box” limits the transparency and interpretability of study results.^34^

Lastly, the occurrence of thromboembolic events in anticoagulated AF patients is influenced by various factors. Our study did not consider determinants beyond demographics, such as OAC under treatment and poor adherence, which could potentially influence patient outcomes.^35^ These elements may be crucial in understanding the risk profile of AF patients on anticoagulation therapy.

## Conclusion

The phenotypic profiles of patients with AF prescribed OACs identified using hierarchical cluster analysis are associated with distinct residual thromboembolic risks and related outcomes. This approach has the potential to enhance patient risk-stratification and holistic approaches to management.

## Supplementary Information

Below is the link to the electronic supplementary material.

Supplementary file1 (DOCX 124 KB)

## Data Availability

The data of this study are available from the corresponding author upon reasonable request.

## Abbreviations

AF: Atrial fibrillation
ACEI: Angiotensin-converting enzyme inhibitors
BMI: Body mass index
BP: Blood pressure
CAD: Coronary artery disease
CHF: Congestive heart failure
CI: Confidence interval
HR: Hazard ratio
OACs: Oral anticoagulants
PAF: Paroxysmal AF
TIA: Transient ischaemic attack
VKA: Vitamin K antagonists

## Declarations:

### Disclaimer:

The authors are solely responsible for the design and conduct of this study, all study analyses, the drafting and editing of the manuscript, and its final contents.

## References

[1] **Hart RG, Pearce LA, Aguilar MI.** Meta-analysis: Antithrombotic therapy to prevent stroke in patients who have nonvalvular atrial fibrillation. Ann Intern Med. 2007;146(12):857. 10.7326/0003-4819-146-12-200706190-00007. 17577005 10.7326/0003-4819-146-12-200706190-00007

[2] **Lip GYH, Laroche C, Dan G-A, et al.** ‘Real-World’ antithrombotic treatment in atrial fibrillation: The EORP-AF pilot survey. Am J Med. 2014;127(6):519-529.e1. 10.1016/j.amjmed.2013.12.022. 24486284 10.1016/j.amjmed.2013.12.022

[3] **Shantsila A, Lip GYH, Lane DA.** Contemporary management of atrial fibrillation in primary and secondary care in the UK: the prospective long-term AF-GEN-UK Registry. EP Eur. 2023;25(2):308-317. 10.1093/europace/euac153. 10.1093/europace/euac153 PMC9934988 36037021

[4] **Ding WY.** Residual stroke risk in atrial fibrillation. Arrhythmia Electrophysiol Rev. 2021;10(3):147–153. 10.15420/aer.2021.34. 10.15420/aer.2021.34 PMC8576486 34777818

[5] **Ding WY, Lane DA, Gupta D, Huisman MV, Lip GYH.** Incidence and risk factors for residual adverse events despite anticoagulation in atrial fibrillation: Results from phase II/III of the GLORIA‐AF Registry. J Am Heart Assoc. 2022;11(15). 10.1161/JAHA.122.026410. 10.1161/JAHA.122.026410 PMC9375480 35876418

[6] **Romiti GF, Proietti M, Bonini N, et al.** Clinical complexity domains, anticoagulation, and outcomes in patients with atrial fibrillation: A Report from the GLORIA-AF Registry Phase II and III. Thromb Haemost. 2022;122(12):2030-2041. 10.1055/s-0042-1756355. 36037828 10.1055/s-0042-1756355

[7] **Grymonprez M, Petrovic M, De Backer TL, Steurbaut S, Lahousse L.** The impact of polypharmacy on the effectiveness and safety of non-vitamin K antagonist oral anticoagulants in patients with atrial fibrillation. Thromb Haemost. 2024;124(02):135-148. 10.1055/s-0043-1769735. 37369234 10.1055/s-0043-1769735 PMC10824584

[8] **Treewaree S, Lip GYH, Krittayaphong R.** Non-vitamin K antagonist oral anticoagulant, warfarin, and ABC pathway adherence on hierarchical outcomes: win ratio analysis of the COOL-AF registry. Thromb Haemost. 2024;124(01):069-079. 10.1055/s-0043-1772773. 10.1055/s-0043-1772773 37625457

[9] **Nielsen JC, Lin YJ, de Oliveira Figueiredo MJ, et al.** European Heart Rhythm Association (EHRA)/Heart Rhythm Society (HRS)/Asia Pacific Heart Rhythm Society (APHRS)/Latin American Heart Rhythm Society (LAHRS) expert consensus on risk assessment in cardiac arrhythmias: Use the right tool for the right outcome,. Europace. 2020;22(8):1147-1148. 10.1093/europace/euaa065. 32538434 10.1093/europace/euaa065 PMC7400488

[10] **Ahmad T, Pencina MJ, Schulte PJ, et al.** Clinical implications of chronic heart failure phenotypes defined by cluster analysis. J Am Coll Cardiol. 2014;64(17):1765-1774. 10.1016/j.jacc.2014.07.979. 25443696 10.1016/j.jacc.2014.07.979 PMC4254424

[11] **Ogawa H, An Y, Nishi H, et al.** Characteristics and clinical outcomes in atrial fibrillation patients classified using cluster analysis: the Fushimi AF Registry. EP Eur. 2021;23(9):1369-1379. 10.1093/europace/euab079. 10.1093/europace/euab079 33930126

[12] **Inohara T, Shrader P, Pieper K, et al.** Association of of atrial fibrillation clinical phenotypes with treatment patterns and outcomes. JAMA Cardiol. 2018;3(1):54. 10.1001/jamacardio.2017.4665. 29128866 10.1001/jamacardio.2017.4665 PMC5833527

[13] **Krittayaphong R, Treewaree S, Wongtheptien W, Kaewkumdee P, Lip GYH.** Clinical phenotype classification to predict risk and optimize the management of patients with atrial fibrillation using the Atrial Fibrillation Better Care (ABC) pathway: a report from the COOL-AF registry. QJM An Int J Med. Published online October 3, 2023. 10.1093/qjmed/hcad219. 10.1093/qjmed/hcad219 37788118

[14] **Huisman M V., Lip GYH, Diener HC, et al.** Design and rationale of Global Registry on Long-Term Oral Antithrombotic Treatment in Patients with Atrial Fibrillation: A global registry program on long-term oral antithrombotic treatment in patients with atrial fibrillation. Am Heart J. 2014;167(3):329-334. 10.1016/j.ahj.2013.12.006. 24576516 10.1016/j.ahj.2013.12.006

[15] **van der Wall SJ, Lip GYH, Teutsch C, et al.** Low bleeding and thromboembolic risk with continued dabigatran during cardiovascular interventions: the GLORIA-AF study. Eur J Intern Med. 2021;91:75-80. 10.1016/j.ejim.2021.05.020. 34120814 10.1016/j.ejim.2021.05.020

[16] **Romiti GF, Proietti M, Corica B, et al.** Implications of clinical risk phenotypes on the management and natural history of atrial fibrillation: A report from the GLORIA‐AF. J Am Heart Assoc. 2023;12(20). 10.1161/JAHA.123.030565. 10.1161/JAHA.123.030565 PMC10757542 37815118

[17] **Schulman S, Kearon C.** Definition of major bleeding in clinical investigations of antihemostatic medicinal products in non‐surgical patients. J Thromb Haemost. 2005;3(4):692-694. 10.1111/j.1538-7836.2005.01204.x. 15842354 10.1111/j.1538-7836.2005.01204.x

[18] **Inohara T, Piccini JP, Mahaffey KW, et al.** A cluster analysis of the Japanese multicenter outpatient registry of patients with atrial fibrillation. Am J Cardiol. 2019;124(6):871-878. 10.1016/j.amjcard.2019.05.071. 31350002 10.1016/j.amjcard.2019.05.071

[19] **Romero-Corral A, Montori VM, Somers VK, et al.** Association of bodyweight with total mortality and with cardiovascular events in coronary artery disease: a systematic review of cohort studies. Lancet. 2006;368(9536):666-678. 10.1016/S0140-6736(06)69251-9. 16920472 10.1016/S0140-6736(06)69251-9

[20] **Benincasa G, Costa D, Infante T, Lucchese R, Donatelli F, Napoli C.** Interplay between genetics and epigenetics in modulating the risk of venous thromboembolism: A new challenge for personalized therapy. Thromb Res. 2019;177:145-153. 10.1016/j.thromres.2019.03.008. 30903874 10.1016/j.thromres.2019.03.008

[21] **Coral DE, Fernandez-Tajes J, Tsereteli N, et al.** A phenome-wide comparative analysis of genetic discordance between obesity and type 2 diabetes. Nat Metab. 2023;5(2):237-247. 10.1038/s42255-022-00731-5. 36703017 10.1038/s42255-022-00731-5 PMC9970876

[22] **Corica B, Romiti GF, Proietti M, et al.** Clinical outcomes in metabolically healthy and unhealthy obese and overweight patients with atrial fibrillation: findings from the GLORIA-AF Registry. Mayo Clin Proc. Published online August 2023. 10.1016/j.mayocp.2023.07.013. 10.1016/j.mayocp.2023.07.013 37632485

[23] **Guo Y, Wang H, Tian Y, Wang Y, Lip GYH.** Multiple risk factors and ischaemic stroke in the elderly Asian population with and without atrial fibrillation. Thromb Haemost. 2016;115(01):184-192. 10.1160/TH15-07-0577. 26322338 10.1160/TH15-07-0577

[24] **Kodani E, Atarashi H, Inoue H, et al.** Impact of blood pressure control on thromboembolism and major hemorrhage in patients with nonvalvular atrial fibrillation: A subanalysis of the J‐RHYTHM registry. J Am Heart Assoc. 2016;5(9). 10.1161/JAHA.116.004075. 10.1161/JAHA.116.004075 PMC5079049 27620886

[25] **Ishii M, Ogawa H, Unoki T, et al.** Relationship of hypertension and systolic blood pressure with the risk of stroke or bleeding in patients with atrial fibrillation: The Fushimi AF registry. Am J Hypertens. 2017;30(11):1073-1082. 10.1093/ajh/hpx094. 28575205 10.1093/ajh/hpx094

[26] **Vemulapalli S, Hellkamp AS, Jones WS, et al.** Blood pressure control and stroke or bleeding risk in anticoagulated patients with atrial fibrillation: Results from the ROCKET AF Trial. Am Heart J. 2016;178:74-84. 10.1016/j.ahj.2016.05.001. 27502854 10.1016/j.ahj.2016.05.001

[27] **Lip GYH, Frison L, Grind M.** Effect of hypertension on anticoagulated patients with atrial fibrillation. Eur Heart J. 2007;28(6):752-759. 10.1093/eurheartj/ehl504. 17289744 10.1093/eurheartj/ehl504

[28] **Arima H, Hart RG, Colman S, et al.** Perindopril-based blood pressure–lowering reduces major vascular events in patients with atrial fibrillation and prior stroke or transient ischemic attack. Stroke. 2005;36(10):2164-2169. 10.1161/01.STR.0000181115.59173.42. 16141420 10.1161/01.STR.0000181115.59173.42

[29] **Romiti GF, Pastori D, Rivera-Caravaca JM, et al.** Adherence to the ‘Atrial Fibrillation Better Care’ pathway in patients with atrial fibrillation: Impact on clinical outcomes—A systematic review and meta-analysis of 285,000 patients. Thromb Haemost. 2022;122(03):406-414. 10.1055/a-1515-9630. 34020488 10.1055/a-1515-9630

[30] **Romiti GF, Proietti M, Bonini N, et al.** Adherence to the Atrial Fibrillation Better Care (ABC) pathway and the risk of major outcomes in patients with atrial fibrillation: A post-hoc analysis from the prospective GLORIA-AF Registry. eClinicalMedicine. 2023;55:1–10. 10.1016/j.eclinm.2022.101757. 10.1016/j.eclinm.2022.101757 PMC9706520 36457650

[31] **Romiti GF, Guo Y, Corica B, Proietti M, Zhang H, Lip GYH.** Mobile Health-Technology-Integrated care for atrial fibrillation: A win ratio analysis from the mAFA-II randomized clinical trial. Thromb Haemost. 2023;123(11):1042-1048. 10.1055/s-0043-1769612. 37247623 10.1055/s-0043-1769612

[32] **Chao T-F, Joung B, Takahashi Y, et al.** 2021 Focused update consensus guidelines of the asia pacific heart rhythm society on stroke prevention in atrial fibrillation: Executive summary. Thromb Haemost. 2022;122(01):020-047. 10.1055/s-0041-1739411. 10.1055/s-0041-1739411 PMC8763451 34773920

[33] **Romiti GF, Corica B, Proietti M, et al.** Patterns of oral anticoagulant use and outcomes in Asian patients with atrial fibrillation: a post-hoc analysis from the GLORIA-AF Registry. eClinicalMedicine. 2023;63:102039. 10.1016/j.eclinm.2023.102039. 10.1016/j.eclinm.2023.102039 PMC10518516 37753446

[34] **Napoli C, Benincasa G, Donatelli F, Ambrosio G.** Precision medicine in distinct heart failure phenotypes: Focus on clinical epigenetics. Am Heart J. 2020;224:113-128. 10.1016/j.ahj.2020.03.007. 32361531 10.1016/j.ahj.2020.03.007

[35] **Paciaroni M, Agnelli G, Caso V, et al.** Causes and risk factors of cerebral ischemic events in patients with atrial fibrillation treated with non–vitamin K antagonist oral anticoagulants for stroke prevention. Stroke. 2019;50(8):2168-2174. 10.1161/STROKEAHA.119.025350. 31234756 10.1161/STROKEAHA.119.025350

