# Supplementary material for: Residual Risks of Thrombotic Complications in Anticoagulated Patients with Atrial Fibrillation: A Cluster Analysis Approach from the GLORIA-AF Registry
Source: J Gen Intern Med. 2024 Sep 25;40(6):1227–37. doi: 10.1007/s11606-024-09045-6 (PMC12045919; doi:10.1007/s11606-024-09045-6)
Supplement: Supplementary file 1 — Supplementary file1 (DOCX 124 KB) [file 11606_2024_9045_MOESM1_ESM.docx]

**Supplementary Materials**

**Supplementary Figure 1. Study Flow Diagram**


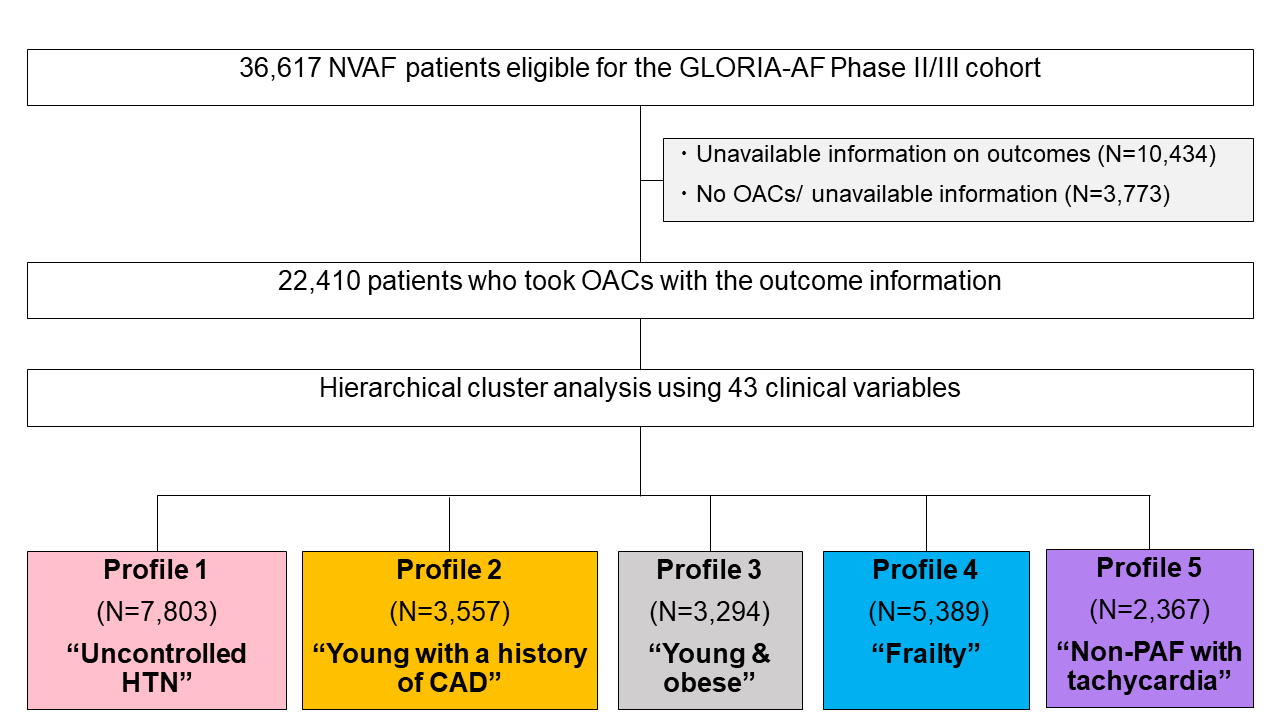


CAD, coronary artery disease; GLORIA-AF, Global Registry on Long-Term Oral Anti-thrombotic Treatment in Patients With Atrial Fibrillation; HTN, hypertension; NVAF, nonvalvular atrial fibrillation; OACs, oral anticoagulants; PAF, paroxysmal atrial fibrillation

**Supplementary Table 1. Average Silhouette Width and Cluster Distribution for Different Numbers of Profiles**

| Numbers of profiles | Average silhouette width | Cluster distribution | | | | | |
| --- | --- | --- | --- | --- | --- | --- | --- |
|  |  | 1 | 2 | 3 | 4 | 5 | 6 |
| 3 | 0.15 | 13,727 | 3,294 | 5,389 | - | - | - |
| 4 | 0.18 | 11,360 | 3,294 | 5,389 | 2,367 | - | - |
| 5 | 0.16 | 7,803 | 3,557 | 3,294 | 5,389 | 2,367 | - |
| 6 | 0.14 | 7,803 | 3,557 | 2,041 | 5,389 | 2,367 | 1,253 |
